# Tissue-Dependent Variation Profiles of Tea Quality-Related Metabolites in New Shoots of Tea Accessions

**DOI:** 10.3389/fnut.2021.659807

**Published:** 2021-04-30

**Authors:** Hiroto Yamashita, Hideyuki Katai, Toshiyuki Ohnishi, Akio Morita, Sanjib Kumar Panda, Takashi Ikka

**Affiliations:** ^1^Faculty of Agriculture, Shizuoka University, Shizuoka, Japan; ^2^United Graduate School of Agricultural Science, Gifu University, Gifu, Japan; ^3^Tea Research Center, Shizuoka Prefectural Research Institute of Agriculture and Forestry, Kikugawa, Shizuoka, Japan; ^4^Institute for Tea Science, Shizuoka University, Shizuoka, Japan; ^5^Department of Biochemistry, School of Life Sciences, Central University of Rajasthan, Rajasthan, India

**Keywords:** tea, volatiles, catechins, theanine, genetic variation

## Abstract

Several metabolites define tea quality in new tea shoots composed of leaf and stem. To improve tea quality for breeding, it is important to understand the tissue-dependent genetic mechanisms and metabolic network responsible for the profile of tea quality-related metabolites. We analyzed the volatiles and specialized metabolites as the tea quality-related metabolites in leaves and stems of new shoots in 30 tea accessions to understand the tissue variation and network between tea quality-related metabolites. Our results provided the tissue-dependent variation network in the tea quality-related metabolites, including volatiles in new leaves and stems in tea accessions. Each volatile content in tea accessions showed the coefficient of variation ranging from 58.7 to 221.9% and 54.2 to 318.3% in new leaves and new stems, respectively. The accumulation pattern of tea quality-related metabolites in new leaves and stems varied depending on the accession. When comparing tea genetic populations, the profile of tea quality-related metabolites of new leaves, but not new stems, was the key to distinguishing tea genetic populations by chemical indicators. We described the network between tea quality-related metabolites, especially the dense network in new leaves. These results also will provide the key information for metabolic engineering and the selection of breeding materials in tea plants based on the tea quality-related metabolites and aid in understanding their molecular mechanisms and network of metabolic variation.

## Introduction

Tea is a popular beverage, and cultivated areas, as well as tea production, are increasing annually worldwide (http://faostat.fao.org/). In general, tea quality is defined by several chemical components, such as color, taste, and aroma, which are affected by the metabolites in the leaves and stems. Tea leaves contain specialized metabolites, such as theanine, which is an amino acid, caffeine, which is a purine alkaloid, and catechins, which are flavonoids, which notably affect tea quality. Amino acids in tea, especially theanine, are responsible for the “umami” taste (1), whereas catechins and caffeine in tea contribute to its astringent properties (2). Epigallocatechin gallate (EGCG), the main catechin in tea, possesses strong bioactivities, such as antioxidant (3) and anticancer (4). Volatiles are fundamental for tea odor and aroma. More than 600 volatile compounds have been identified in the tea aroma (5). Furthermore, the volatile composition of tea depends on the materials and processing methods. Most volatiles in fresh tea leaves are glycosidically bound precursors that are released by hydrolases, such as β-primeverosidase, during the tea manufacturing process of withering and rolling (6–8). There are great variations in aromatic volatiles among various kinds of tea, especially green tea, oolong, and black tea, owing to the differences in tea cultivars and processing methods, resulting in variations in aromatic characteristics (9). However, basic information for breeding on their accumulation variation of tea quality-related metabolites, especially volatiles, is limited, and there have also been limited studies on inter-component networks in tea.

Most tea plants [*Camellia sinensis* (L.) O. Kuntze] grown worldwide originated from China and/or India, and they are roughly divided into two varieties, *sinensis* and *assamica*, respectively. Their genetic diversity levels are also high (10). Assam hybrids, resulting from a cross between var. *sinensis* and var. *assamica*, are mainly utilized as a cultivar of black tea and for improved cold-tolerance breeding, especially in Japan. However, especially in Japan, a few elite cultivars are used as breeding materials, resulting in limited genetic diversity among breeding populations. Because high-quality progeny generally results from crossing high-quality parents (i.e., Japanese accession “Yabukita” is the most popular green tea cultivar in Japan), breeders tend to give the highest priority to such breeding combinations (11). Therefore, it is necessary to increase the genetic diversity of breeding populations. Unfortunately, most tea genetic resources have limited information on agronomic traits for breeding, such as metabolites content, disease resistance, and yield.

For green tea, especially Japanese green tea, the first crop (or flush; harvest at spring) is the highest quality and corresponds to the best harvesting season. The price of tea tends to be proportional to the nitrogen contents, which depends on the amino acid levels (12). In addition, the price of tea per kilogram is highest for the first crop because of the higher quality, and it is more than twice the price of the second crop. Even in black tea, the aroma differs greatly between the first and the second crop, and their characteristics also change depending on the cultivar and the environment of cultivation management (13). To improve tea quality by breeding, it is important to understand the mechanisms and network responsible for metabolite-related characteristics in tea new shoots.

Metabolomic approaches have been used to dissect large-scale metabolic composition and regulation networks in plants, generating new information that could be potentially applied in crop breeding for improved metabolic traits (14). In addition, the metabolic correlation, as well as gene co-expression, approaches have highlighted some properties in several plants (15–17). Fukushima et al. (16) demonstrated that differences in tissues in *Arabidopsis* affect changes in the topology of metabolic correlation networks. Revealing tissue-dependent metabolic networks in tea plants is also essential to achieve metabolic engineering in new tea shoots composed of leaf and stem.

Here, we analyzed the volatiles and specialized metabolites as the tea quality-related metabolites in leaves and stems of new shoots in 30 tea accessions to understand the variation and network between these metabolites. We observed the clear differences in genetic variations of metabolic profile in leaves and stems. Furthermore, network-based analysis (18, 19) identified further differences and relationships of tea quality-related metabolic profiles in leaves and stems.

## Materials and Methods

### Plant Materials

Thirty tea accessions of var. *sinensis* and Assam hybrids, which are considered to have different genetic backgrounds and origins (Supplementary Table 1), were selected in this study. In the first crop season of 2017, new shoots of each accession at the same developmental stage were plucked around noon at the Tea Research Center, Shizuoka Prefectural Research Institute of Agriculture and Forestry, Kikugawa, Shizuoka, Japan. The tea ridges were managed to match the status using conventional methods (such as fertilization, skiffing, deep-pruning, etc.) optimized for Japanese green tea cultivation, although their tree ages were slightly different. Nitrogen fertilizer was applied at 400 kg-N ha^−1^ year^−1^. In this study, new shoots were defined as those with four leaves developed. After being plucked, tea shoots were immediately separated into new leaves and stems (Supplementary Figure 1), and then, they were stored at −80°C until the volatile measurement. The rest of the samples were freeze-dried, ground into a fine powder, and stored at room temperature within a desiccator until measurement of catechins, caffeine, and amino acids. Each accession was measured with one to three replicates, and the average value was taken as the phenotypic value.

### Volatiles Measurement

Fresh plant tissues (500 mg) were ground into a fine powder under cryogenic conditions with liquid nitrogen and extracted with 3-ml n-pentane:diethyl ether [1:1 (v/v)] for 16 h under dark conditions. Ethyl decanoate (5 nmol), as an internal standard, was also added to the resulting solution. After 16 h, the supernatant underwent solid-phase extraction using InertSep®GC (GL Science Inc., Tokyo, Japan). Anhydrous sodium sulfate was added to the extract for dehydration. The samples were left standing for 10 min and then concentrated to approximately 1 ml under nitrogen gas. The resulting supernatant was stored at −30°C until analyzed by gas chromatography–mass spectrometry (GC-MS). Volatiles were quantified on a gas chromatograph equipped with a mass spectrometer (GCMS-QP2010Plus, Shimadzu, Tokyo, Japan), having an electrospray ionization source. The GC-MS analysis was performed at an injector temperature of 240°C with helium as the carrier gas helium at a constant pressure of 108 kPa. A Stabilwax column (60 m × 250 μm × 0.25 μm, GL Science Inc., Tokyo, Japan) was used with an initial temperature of 40°C for 2 min, a ramp of 4°C/min to 240°C and then hold at 240°C for 25 min. The MS analyses were carried out in SCAN mode (mass range *m/z* 30–280). Data analyses were performed using LabSolutions GCSolution ver. 2.72 (Shimadzu, Tokyo, Japan). The 20 volatiles, 1-penten-3-ol, (*Z*)-2-penten-1-ol, (*Z*)-3-hexen-1-ol, linalool oxideI, linalool oxideII, linalool oxide III, linalool oxide, linalool, methyl salicylate, nerol, geraniol, benzyl alcohol, 2-phenylethanol, β-ionone, (*Z*)-jasmone, 8-hydroxy linalool (8-OH linalool), methyl anthranilate, jasmin lactone, methyl jasmonate, indole, and comarin, were used as standards and quantified.

### Catechin and Caffeine Measurements

Catechins and caffeine contents were measured according to the methods described by Horie et al. (20) and Yamashita et al. (21). Dry ground samples (25 mg) were added to 5-ml 50% (v/v) acetonitrile and shaken (130 strokes per minute) for 60 min at room temperature for the extraction. After centrifugation (2,000 × *g*, 15 min, 4°C), the supernatant was collected and then passed through a 0.45-μm polytetrafluoroethylene filter (ADVANTEC, Tokyo, Japan). The resulting solution was stored at −30°C until analyzed by high-performance liquid chromatography (HPLC). The HPLC system consisted of Shimadzu (Tokyo, Japan) in the following: two LC-10ADvp pumps, D6U-14A degasser, CTO-20AC column oven, SPD-M20A prominence photodiode array detector, SCL-10Avp system controller, and SIL-10ADvp autosampler. HPLC condition was used as following: injection volume, 5 μl; column, 75 mm × 4.6 mm × 2.6 μm SunShell C18 column (ChromaNik Technologies Inc., Osaka, Japan); the temperature of column oven, 40°C; photodiode array detector, 190–400 nm. Eluent A [1,909:90:1 ml (v/v/v), ultra-pure water:acetonitrile: 85% phosphoric acid] and eluent B [999:1,000:1 mL (v/v/v), ultra-pure water:acetonitrile: 85% phosphoric acid] were used as the mobile phases at a flow rate of 1.0 ml/min. The elution was performed with the following gradient: initial concentration of 10% B, followed by a 2.5 min hold at 10% B, a 1.5-min linear gradient from 10 to 30% B, a 1.0-min hold at 30% B, a 2.5-linear gradient 30 to 80% B, a 2.5-min hold at 80% B, a 1.0-min gradient from 80 to 10% B, and a final concentration of 10% B for 4.0 min. The solution of this mobile phase was eluted for 15 min per sample. The seven catechins, (+)-gallocatechin (GC), (+)-catechin (C), (–)-epicatechin (EC), (–)-epigallocatechin (EGC), (–)-catechin gallate (CG), (–)-epicatechin gallate (ECG), and (–)-epigallocatechin gallate (EGCG), and caffeine were used as standards and quantified.

### Free Amino Acids Measurement

The free amino acids (FAAs) contents were measured according to the method described by Goto et al. (22) and Yamashita et al. (21). Dry ground samples (10 mg) were added to 10-mg polyvinylpolypyrrolidone and 5-ml ultra-pure water and shaken (130 strokes per minute) for 60 min at room temperature for the extraction. After centrifugation (2,000 × *g*, 15 min, 4°C), the supernatant was collected and then passed through a 0.45-μm cellulose acetate filter (ADVANTEC, Tokyo, Japan). The resulting solution was stored at −30°C until analyzed by HPLC. Homoserine, as an internal standard, was added to the resulting solution, and *o-*phthalaldehyde derivatives were analyzed using the HPLC system (Shimadzu, Japan, Tokyo). The HPLC system consisted of Shimadzu (Tokyo, Japan) in the following: two LC-10AT pumps, DGU-20A5R degasser, CTO-10Avp column oven, RF-20A prominence fluorescence detector, SCL-10Avp system controller, and SIL-10AF autosampler. HPLC condition was used as following: injection volume, 5 μl; column, 75 mm × 4.6 mm × 5 μm Ascentis Express C18 column (Sigma-Aldrich, St. Louis, MO, USA); the temperature of column oven, 40°C; excitation wavelength 340 nm; emission wavelength 450 nm. Eluent A [5-mM citrate buffer, pH 6.0 and 5% (v/v) acetonitrile] and eluent B [5-mM citrate buffer, pH 6.0 and 70% (v/v) acetonitrile] were used as the mobile phases at a flow rate of 1.0 ml/min. The elution was performed with the following gradient: initial concentration of 5% B, followed by a 1.6-min linear gradient from 5 to 12% B, a 5.0-min linear gradient from 12 to 22% B, a 1.7-min linear gradient from 22 to 95% B, a 2.2-min hold at 95% B, a 0.5-min linear gradient from 95 to 5% B, a 1.5-min gradient from 5 to 0% B, and a final concentration of 0% B for 1.0 min. The solution of this mobile phase was eluted for 15 min per sample. Nine amino acids, aspartate (Asp), asparagine (Asn), glutamate (Glu), glutamine (Gln), serine (Ser), arginine (Arg), alanine (Ala), theanine, and γ-aminobutyric acid (GABA), were used as standards and quantified.

### Multivariate Analysis

Data were normalized by calculating z-score values among the metabolites. A principal component analysis (PCA) was performed using the R function “prcomp.” A hierarchical cluster analysis was performed based on Ward's method (23) using Euclidean distances and was conducted using the R function “hclust.” A heatmap was visualized using the “heatmap.2” function of the R package gplots ver. 3.0.1.

### Network Analysis

The significance of correlations was determined while correcting for multiple comparisons. Correlations were called significant when false discovery rate < 0.05 using the R package q-value ver. 2.12.0. Only significant correlations were used in the construction of networks. Visualization and a centrality analysis of the network were performed on Cytoscape version 3.5.1.

## Results

### Variation of Volatiles and Tea Specialized Metabolites in New Leaves and Stems

**Figure 1:**
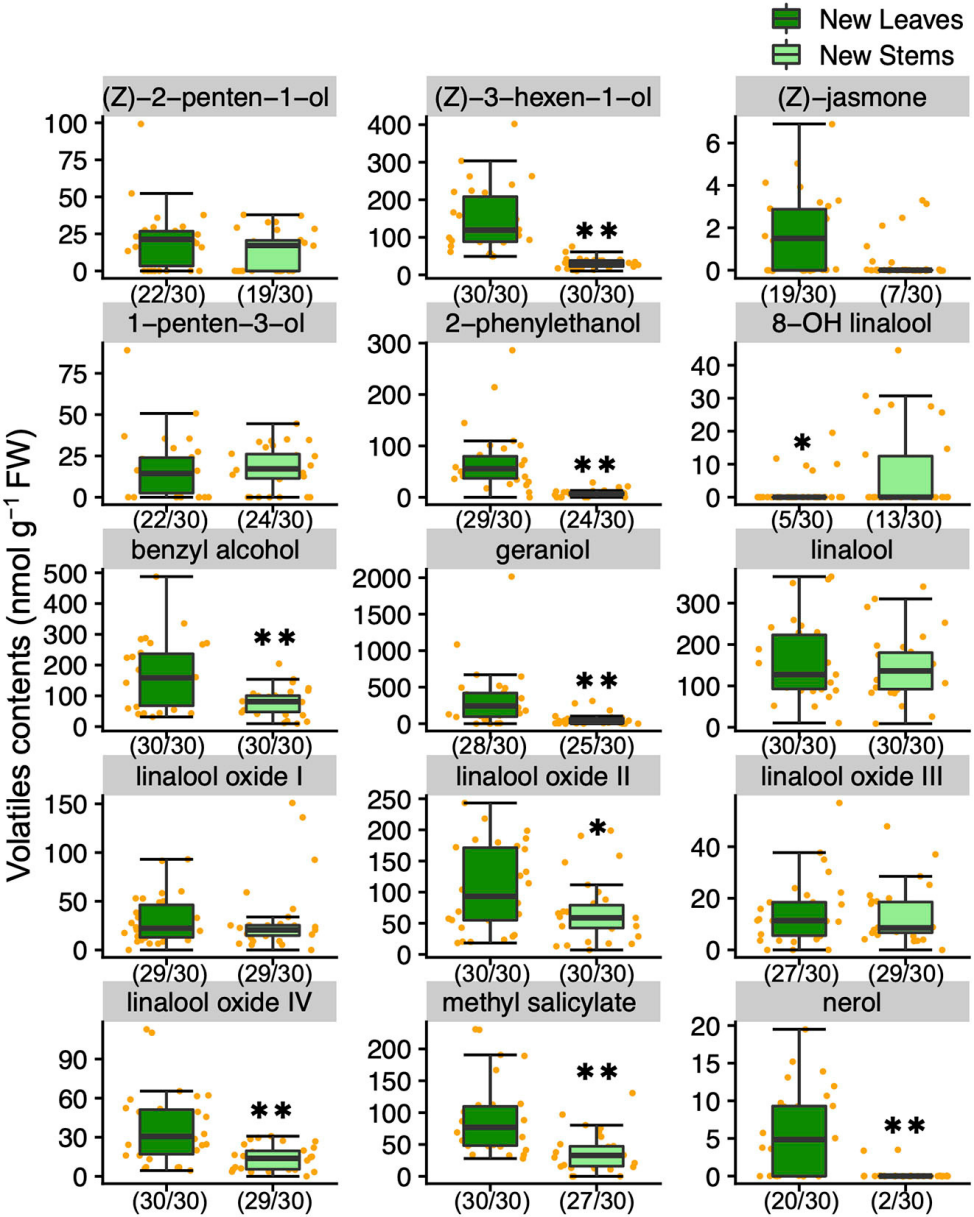
Boxplots of volatiles contents in new leaves and stems of the tea accessions. Single and double asterisks indicate *P* < 0.05 and *P* < 0.01, respectively (Welch's t-test). Orange plots indicate the values of each accession. Left and right numbers in parentheses indicate quantified and analyzed accessions, respectively.

**Figure 2:**
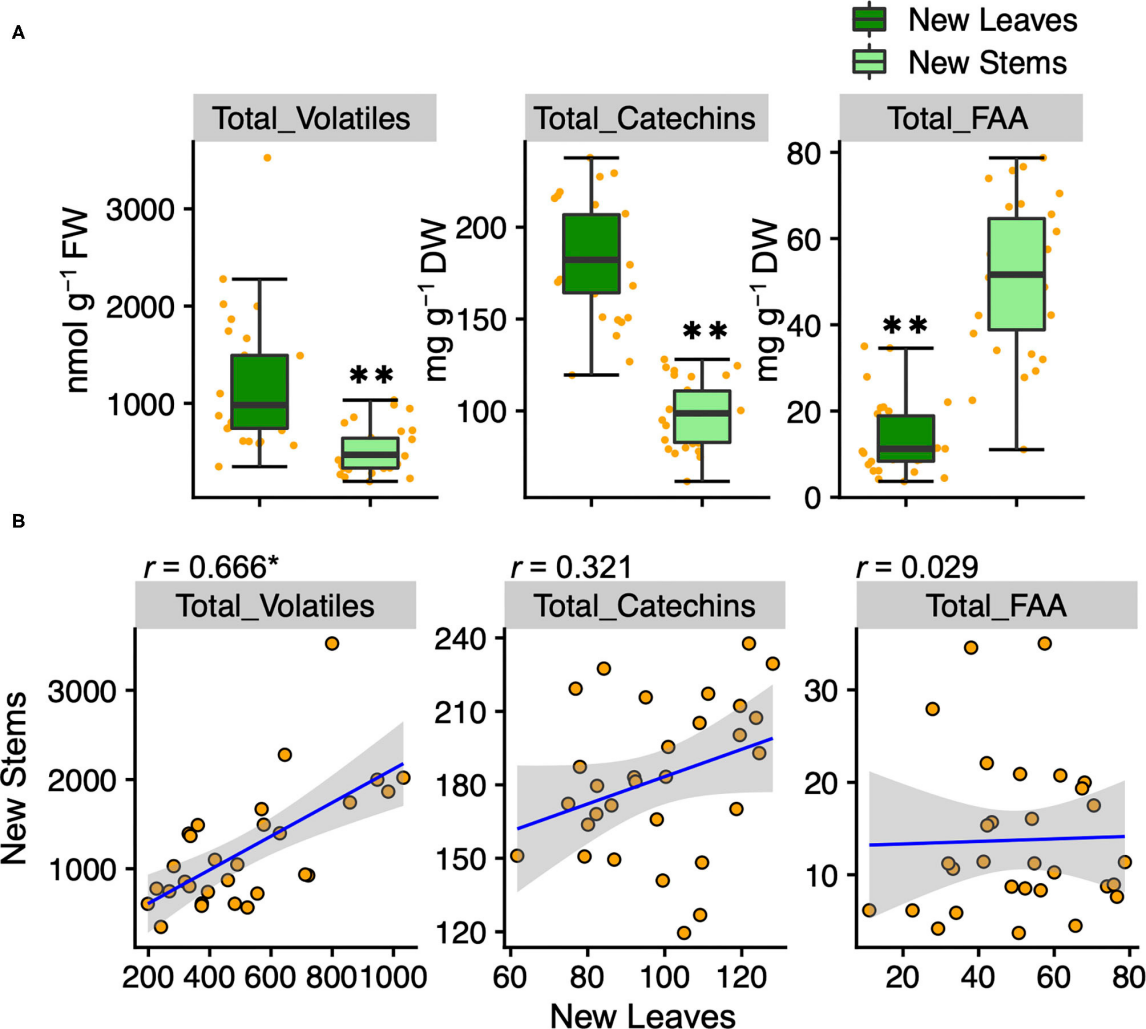
Boxplots **(A)** and correlations **(B)** of total volatiles, catechins, and amino acids contents in new leaves and stems of the tea accessions, respectively. Orange plots indicate the values of each accession. Double asterisks in **(A)** indicate *P* < 0.01 (Welch's *t*-test). Asterisk in **(B)** indicates *P* < 0.01 (test of no correlation).

To understand the variation and network between tea quality-related metabolites, we analyzed the volatiles and specialized metabolites in leaves and stems of new shoots in 30 tea accessions. We quantified 34 metabolites, consisting of 17 volatiles, nine amino acids, seven catechins, and caffeine in new leaves and stems. Each volatile content in tea accessions showed wide variations, with a coefficient of variation (CV) ranging from 58.7 to 221.9% and 54.2 to 318.3% in new leaves and new stems, respectively (Supplementary Table 2). The contents of (Z)-3-hexen-1-ol, linalool oxide II, linalool oxide IV, methyl salicylate, nerol, geraniol, benzyl alcohol, and 2-phenylethanol in new leaves were observed significantly greater distributions than in new stems (Figure 1). The content of only 8-OH linalool in new stems had a significantly greater distribution than in new leaves (Figure 1). The total volatile contents of the accessions ranged from 348.7 to 3,524.2 and 197.7 to 1,032.4 nmol g^−1^ fresh weight (FW) in new leaves and stems, respectively (Supplementary Table 2), and the distribution in new leaves was significantly greater than in new stems (Figure 2A). Total catechins, but not total FAA, showed the same distribution trend (Figure 2A). Furthermore, the total volatiles content was significantly correlated (*r* = 0.666, *P* < 0.01) between new leaves and stems, whereas total catechins and FAA showed no correlations (Figure 2B). In each catechin and FAA, GC, (+) C, EGCG, Asp, Asn, Ser, Arg, and GABA were significantly correlated between new leaves and stems, but theanine, the main FAAs in the tea plant, was not (Supplementary Table 2). In addition, the caffeine contents of the accessions ranged from 14.0 to 63.9 mg g^−1^ dry weight (DW) and 11.9 to 28.8 mg g^−1^ DW in new leaves and stems, respectively (Supplementary Table 2 and Supplementary Figure 2A), and caffeine had a significantly greater distribution in new leaves than in new stems (Supplementary Figure 2A).

**Figure 3:**
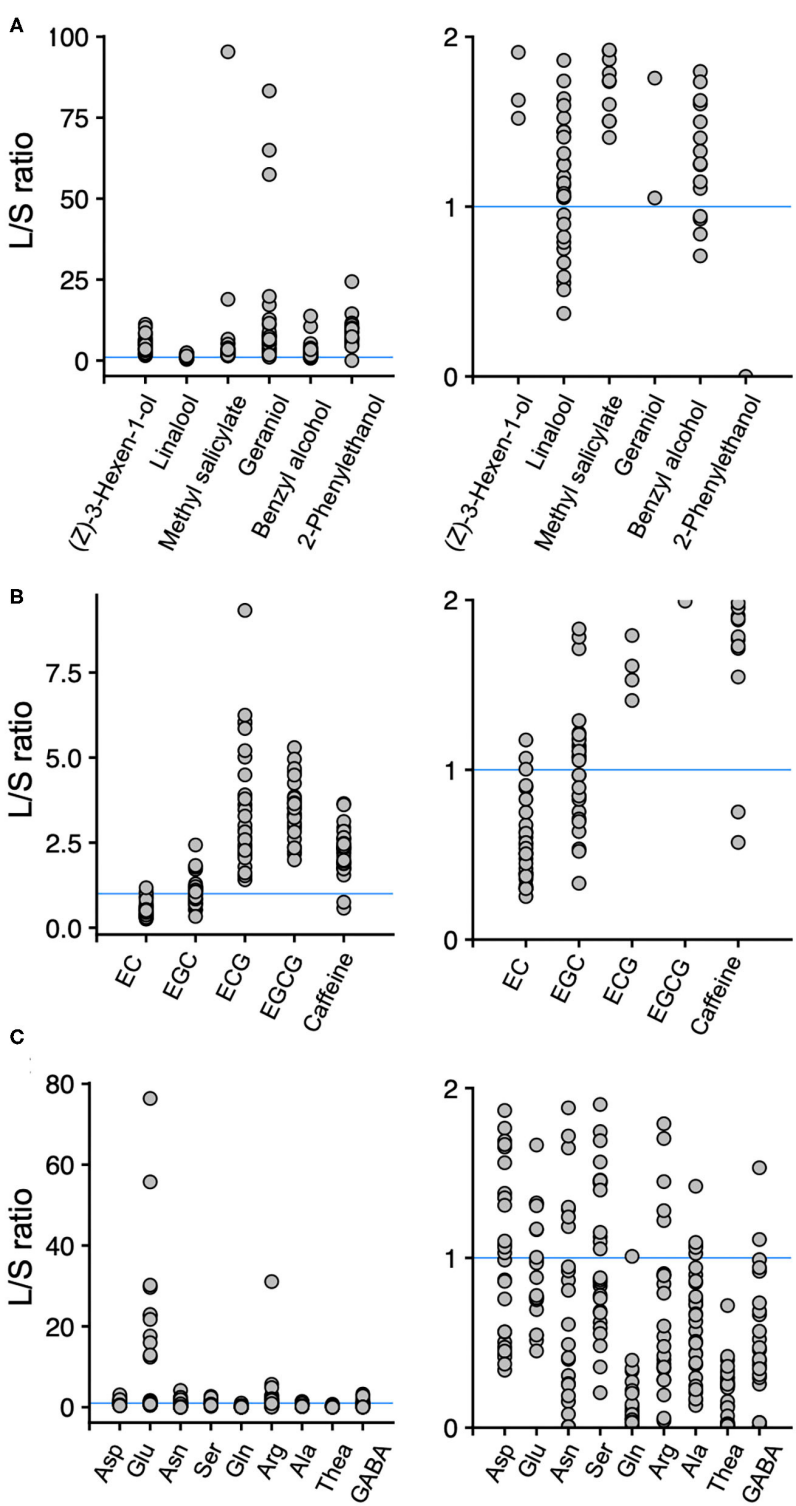
Ratios of the contents of main volatiles **(A)**, main catechins and caffeine **(B)**, and main amino acids **(C)** in new leaves to those in new stems. Each plot represents accessions. Each figure on the right is a close-up of the figures on the left. A leaf/stem (L/S) ratio > 1 indicates that new leaves have a higher content of the component than the new stems, and L/S < 1 indicates that new stems have a higher content of the component than the new leaves in an accession. Blue lines indicate L/S = 1.

To reveal the tissue-accumulation profiles of tea quality-related metabolites, we calculated the ratios of the content of those in new leaves to new stems in the accessions. When the leaf/stem ratio (L/S) value was one, then the contents of the metabolites in new leaves and new stems were the same. Hence, L/S > 1 indicates that the metabolite's content in the new leaves was greater than in the new stems, and L/S < 1 indicates that the metabolite's content in the new leaves was lower than in the new stems. Among the six main volatiles, the L/S values were >1 for (Z)-3-hexen-1-ol, methyl salicylate, geraniol, and 2-phenylethanol in all the accessions, whereas several accessions had L/S values >1 and <1 for linalool and benzyl alcohol, respectively (Figure 3A). Among the main four catechins, L/S > 1 occurred for ECG and EGCG in all the accessions, whereas several accessions had L/S values >1 and <1 for EC and EGC, respectively (Figure 3B). For caffeine, which is synthesized and accumulated in leaves, most of the accessions had L/S values > 1 (Figure 3B). For the nine main amino acids, all the accessions had L/S values of >1 or <1, except for theanine, which always had an L/S value < 1 (Figure 3C).

### Multivariate Analysis of Tea Quality-Related Metabolites

**Figure 4:**
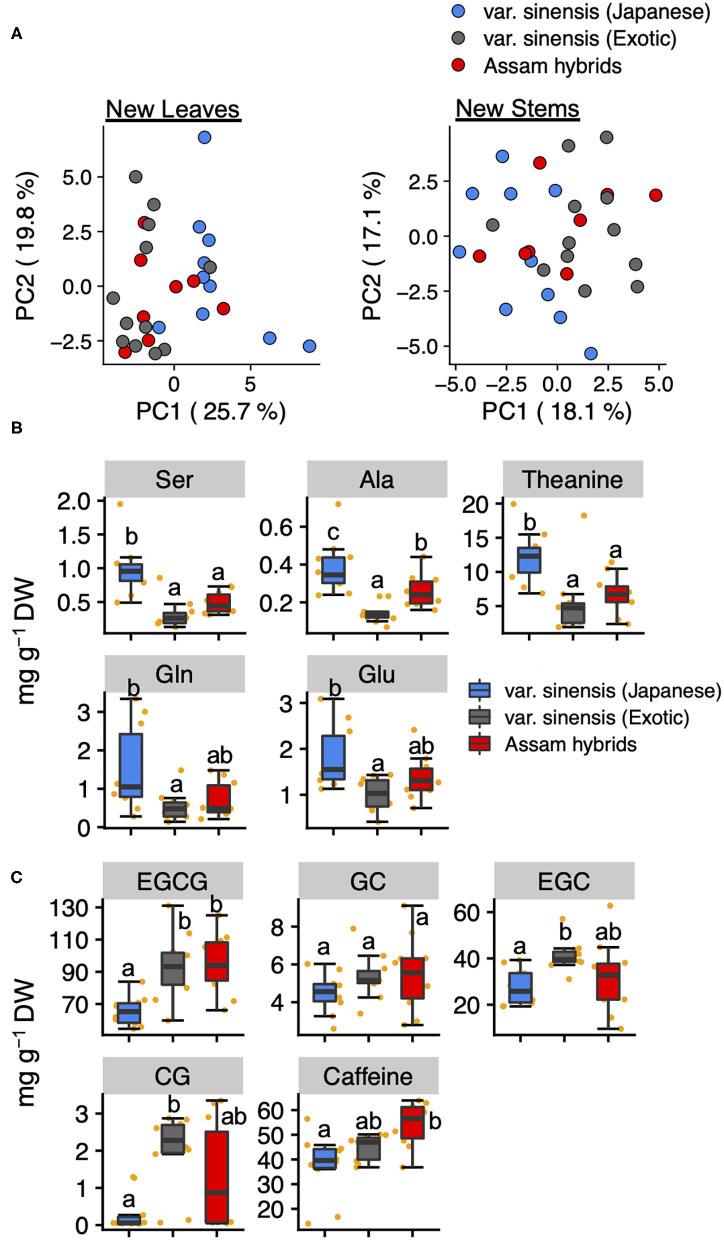
Principal component analysis based on quantified tea quality-related metabolites in new leaves and stems. Plots of PC1 and PC2 in new leaves and stems **(A)**. Different plot colors represent tea genetic populations. Boxplots of the top five metabolites with positive **(B)** and negative **(C)** PC1 factor loading in the PCA of new leaves among genetic populations. Orange plots indicate the values of each accession. Different letters indicate significant differences (Tukey's test, *P* < 0.05).

To estimate the differences in the profiles of tea quality-related metabolites among tea varieties, we compared these quantified metabolites data in new leaves and stems by PCA. A PCA based on metabolites data in new leaves revealed that PC1 could separate Japanese and exotic var. *sinensis* but did not separate Assam hybrids (Figure 4A). Using a factor loading analysis (Supplementary Table 5) based on PC1, we determined that the contents of Ser, Ala, theanine, Glu, and Glu in Japanese var. *sinensis* were significantly higher than in exotic var. *sinensis* (Figure 4B), and the contents of EGCG, EGC, CG, and caffeine in Japanese var. *sinensis* were significantly lower than in exotic var. *sinensis* (Figure 4C). Metabolites in new stems were not separated based on varieties according to each accession's metabolic profile (Figure 4A). These results were also supported by the hierarchical cluster analysis (Supplementary Figure 3).

### Correlation Network-Based Analysis of Tea Quality-Related Metabolites

**Figure 5:**
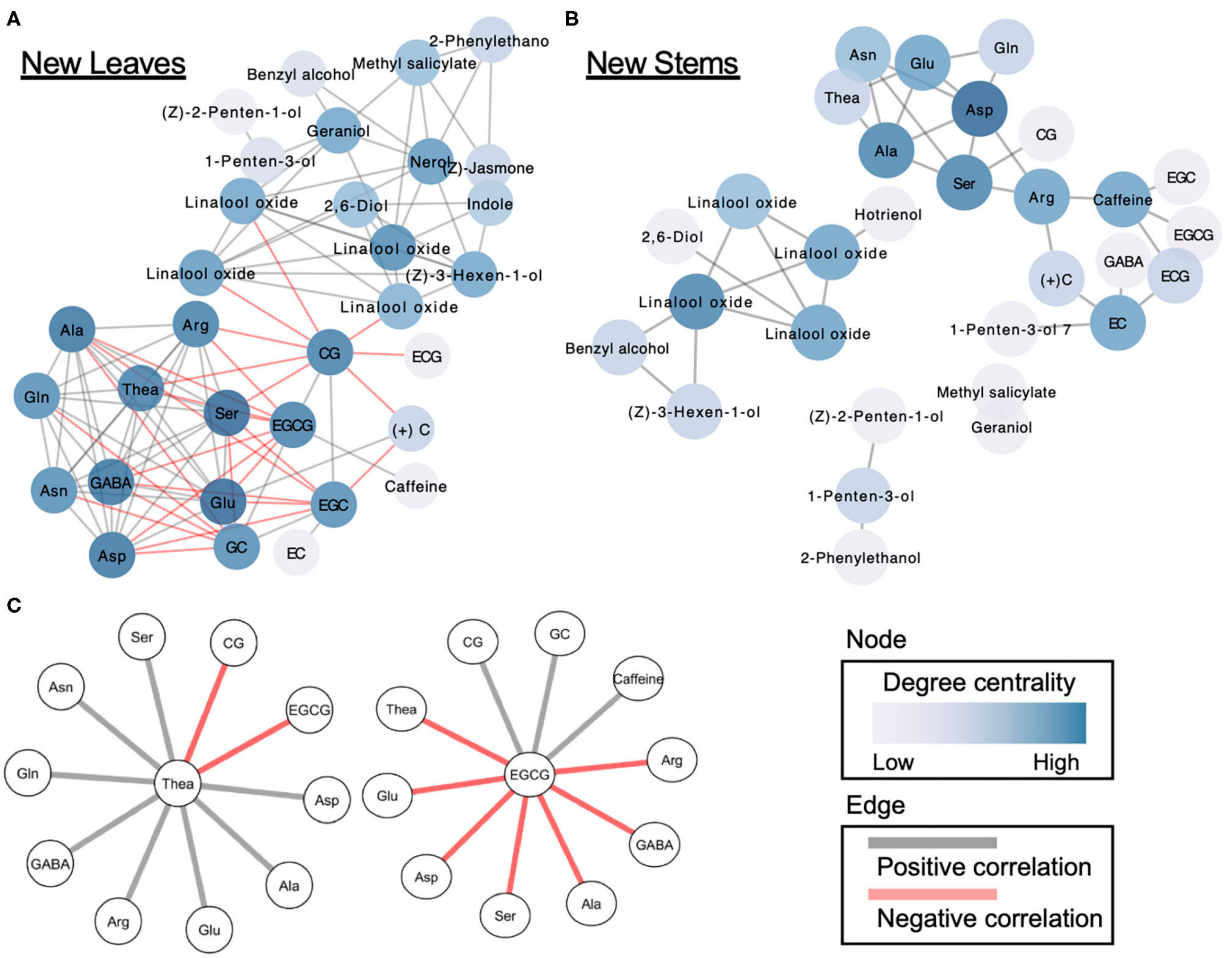
Correlation network-based analysis of tea quality-related metabolites in new leaves **(A)** and stems **(B)**. Node colors indicate degree centrality levels, respectively. Gray and red edge colors indicate positive and negative correlations, respectively. Metabolites forming networks directly with theanine and EGCG **(C)** in new leaves.

Applied correlation analysis of our dataset revealed both positive and negative correlations among metabolites in both new leaves and stems (Supplementary Figure 4). To understand in detail the correlations among the metabolites, we performed a correlation network-based analysis. In new leaves, only one dense network was constructed, whereas in new stems, several sparse networks were constructed (Figures 5A,B). The dense network in new leaves was divided into clusters of volatiles and tea specialized metabolites (Figure 5A). Furthermore, the centrality in the new-leaf derived network is the highest for catechin gallate (Supplementary Table 6). Theanine, the tea specialized and main amino acids, were negatively correlated with EGCG and CG in catechins while positively correlated with many amino acids (Figure 5C). EGCG, the main catechins, was negatively correlated with many amino acids, including theanine, and positively correlated with CG, GC, and caffeine (Figure 5C).

## Discussion

In general, new tea shoots are plucked several times annually from spring to autumn and then manufactured. In particular, new shoots plucked during the first crop season (spring in Japan) are the highest quality among all the tea plucked; therefore, the characteristic chemical composition during the first crop is important. For tea breeding, the characteristics of cultivars are evaluated by the traits of the first crop. However, most tea genetic resources have limited information on agronomic traits for breeding, such as metabolites content. Each tea quality-related metabolite has different characteristics for tea quality; e.g., theanine is responsible for the “umami” taste (1), whereas catechins and caffeine in tea contribute to its astringent properties (2). Hence, the balance of each metabolite is important in leaves and stems, and it is necessary to accurately understand the relationship between each metabolite. In this study, to understand the tissue-dependent variations and networks in the chemical compositions of genetically diverse tea plants, we analyzed volatiles, amino acids, catechins, and caffeine of new shoots in the first crop season across 30 accessions using GC-MS and HPLC.

Each metabolite showed genetic-based variations among accessions in both new leaves and stems (Figure 1, Supplementary Tables 2–4). Main tea volatiles (24), such as (*Z*)-3-hexen-1-ol (green odor), linalool (floral odor), linalool oxides (sweet floral odor), geraniol (floral odor), benzyl alcohol (sweet odor), and 2-phenylethanol (rose-like odor), were quantified in new leaves and stems and showed genetic-based variations (Figure 1, Supplementary Tables 2–4). There has been little progress in breeding for odor, and the evaluation and selection have been limited to the sensory aspect (25). These results would provide the key information for selecting breeding materials in tea plants based on volatiles content. Recently, information on the reference genome (26–28) and single nucleotide polymorphisms (29) of tea accessions has been constructed; therefore, we can expect to develop genetic markers involved in the tea quality-related metabolites by applying genome-wide association study (GWAS) to phenotyping using a larger number of accessions. Actually, in other crop species, the responsible loci have been identified by GWAS based on the content of volatiles (30) and amino acid (31).

The accumulation levels between tissues in new shoots differed depending on the volatiles (Figure 1). In addition, no significant correlations were found between metabolites, such as total catechins and total amino acids, in new leaves and stems (Figure 2). These results revealed the difference between tissue-dependent genetic variations of tea quality-related metabolites in new leaves and stems. Therefore, we calculated the ratios of the contents of tea quality-related metabolites in new leaves to those in new stems among tea accessions. This analysis revealed that the profiles of tea quality-related metabolites in different tissues varied depending on the accession (Figure 3). To clarify the differences among accumulation patterns in tissues at the molecular level, we needed to analyze the expression levels of key genes (such as caffeine synthase) involved in the tea specialized metabolisms because we only conducted a metabolite analysis.

*C. sinensis* is classified into two major varieties, var. *sinensis* and var. *assamica*. In Japan, Assam hybrids, resulting from crosses between var. *sinensis* and var. *assamica*, are mainly utilized as cultivars for breeding and making black tea. The genetic population structure analysis in the current study divided the germplasms into Japanese var. *sinensis* and exotic accessions, and furthermore, the latter could be divided into var. *sinensis* and var. *assamica* (11, 21, 29). In this study, we selected 30 tea accessions from different genetic populations, Japanese var. *sinensis*, exotic var. *sinensis*, and Assam hybrids, which originated from five countries (Japan, India, Sri Lanka, China, and Taiwan) (Supplementary Table 1). To estimate differences in the profiles of tea quality-related metabolites among the tea genetic populations, we compared their metabolites in new leaves and stems in the first crop. When comparing tea genetic populations, total volatiles in new stems and total amino acids in new leaves differed among genetic populations (Supplementary Figures 5A,B). Furthermore, in new leaves, a PCA revealed that PC1 could separate var. *sinensis* (Japanese) and var. *sinensis* (exotic) (Figure 4A). Additionally, the candidates for PC1 were also determined by analyzing factor loading (Supplementary Table 5). The top-ranked metabolites in PC1 factor loading mostly consisted of amino acids and catechins but not volatiles. In new leaves, most amino acid levels (Ser, Ala, theanine, Gln, and Glu) were significantly higher in Japanese var. *sinensis* than in exotic var. *sinensis* and Assam hybrids (Figure 4B), whereas most catechin levels (EGCG, EGC, and CG) were significantly lower in Japanese var. *sinensis* than in exotic var. *sinensis* and Assam hybrids (Figure 4C). However, the total catechin contents in new leaves were similar among tea genetic populations (Supplementary Figure 5). This resulted from the ECG content being significantly higher in Japanese var. *sinensis* than in exotic var. *sinensis* and Assam hybrids (Supplementary Figure 6). Because there have been limited reports comparing specific catechin levels among varieties, these results are important for determining the relationship between genetic background and catechins metabolism. Additionally, the previous study has reported that the Arg content in the first crop of Assam hybrids is lower than in Japanese accessions for green tea (32). Although this previous study focused on the bulk, new shoots, we focused on tissues in both new leaves and stems of the first crop. Our results showed the same genetic structure patterns of Arg content in each tissue (Supplementary Figure 7). Albino tea accessions with etiolated yellow or white leaves have drastically high FAAs, especially Arg contents (33–35). Kobayashi et al. (36) reported that Arg content was drastically also increased in white-leaves generated by dark artificial treatment. Also, in this study, these two white tea accessions showed high amino acid distributions in other green tea accessions (Supplementary Tables 3, 4). In addition, although it is a Chinese tea accession with green-leaf color, “Yunnan large-leaf” had a higher Arg content than the above two Japanese tea accessions with white-leaf color (Supplementary Tables 3, 4). Thus, there is no direct correlation between the white color and Arg content in new shoots, indicating that some other factor directly controls the variation in the Arg content in tea shoots. The caffeine contents in new leaves were highest in Assam hybrids, followed by Japanese var. *sinensis* and then exotic var. *sinensis* (Figure 4C); however, the caffeine contents in the new stems did not differ among tea genetic populations (Supplementary Figure 8). These results were consistent with the report of Takeda (37). These results suggested that the profile of tea quality-related metabolites of new leaves, but not new stems, was the key to distinguishing tea genetic populations by chemical indicators. It may be possible to characterize the differences in metabolite characteristics between varieties by conducting a comprehensive metabolome analysis, such as a non-target metabolome analysis, although we analyzed only the main tea quality-related metabolites in new leaves and stems.

Correlation networks offer a way to investigate patterns of interdependency among metabolites (18, 19). The network plots can provide insights into the functional correlations among metabolites by comparing metabolic correlation networks to known biochemical pathways. We constructed the network using significant correlations (false discovery rate <0.05) identified by multiple tests, and it revealed the correlations among metabolites in new leaves and stems of the first crop (Figure 5). One dense network was constructed in new leaves, whereas several sparse networks were constructed in new stems (Figure 5). Our network analysis revealed that the main tea quality-related metabolites in just the new leaves were correlated, such as volatiles–catechins and amino acids–catechins. However, it is important to reveal the hub metabolites in the correlation networks. Centrality is an important structural attribute of networks (18). Through a centrality analysis (betweenness centrality, closeness centrality, and degree of centrality), we detected several hub metabolites (Figure 5, Supplementary Table 6). Theanine, a major and unique amino acid in tea plants, was metabolized into catechins in leaves with maturity (38, 39). Therefore, we focused on the metabolites involved with theanine in the dense network of new leaves. Which types of catechins are involved in theanine metabolism are limited. Our network analysis revealed that EGCG and CG were negatively correlated with theanine. In addition, EGCG, the main tea catechin, was negatively correlated with many amino acids, including theanine. In addition to theanine, close correlations between other amino acids and catechin metabolism were clarified by this network analysis. Our network analysis showed that there was a negative correlation between theanine and catechin accumulation levels, even with genetic differences, regardless of the maturation of the leaves. These suggest that the metabolic relationship between theanine and catechins is regulated at the molecular genetic level.

## Conclusion

This study provides new information on the tissue-dependent variation network in the tea quality-related metabolites, including volatiles in new leaves and stems of the first crop in tea accessions. The accumulation pattern of tea quality-related metabolites in different tissues varied depending on the accession. When comparing tea genetic populations, the profile of tea quality-related metabolites of new leaves, but not new stems, was the key to distinguishing tea genetic populations by chemical indicators. We described the network between tea quality-related metabolites, especially the dense network in new leaves. These results also will provide the key information for metabolic engineering and the selection of breeding materials in tea plants based on the tea quality-related metabolites and aid in understanding their molecular mechanisms and network of metabolic variation. By using the latest information of reference genome and single nucleotide polymorphisms markers in tea accessions, we can expect to develop genetic markers involved in the tea quality-related metabolites by applying GWAS to phenotyping using a larger number of accessions.

## Data Availability Statement

The original contributions presented in the study are included in the article/Supplementary Material, further inquiries can be directed to the corresponding author/s.

## Author Contributions

HY, TO, and TI performed the experiments. HK managed the tea accessions. HY performed data visualization. HY, SP, AM, and TI designed the study and wrote the manuscript. SP and TI sourced funding. All authors read and approved the manuscript.

## Conflict of Interest

The authors declare that the research was conducted in the absence of any commercial or financial relationships that could be construed as a potential conflict of interest.
