# Supplementary material for: Tissue-Dependent Variation Profiles of Tea Quality-Related Metabolites in New Shoots of Tea Accessions
Source: Front Nutr. 2021 Apr 30;8:659807. doi: 10.3389/fnut.2021.659807 (PMC8119633; doi:10.3389/fnut.2021.659807)
Supplement: Supplementary file 1 [file Data_Sheet_1.PDF]

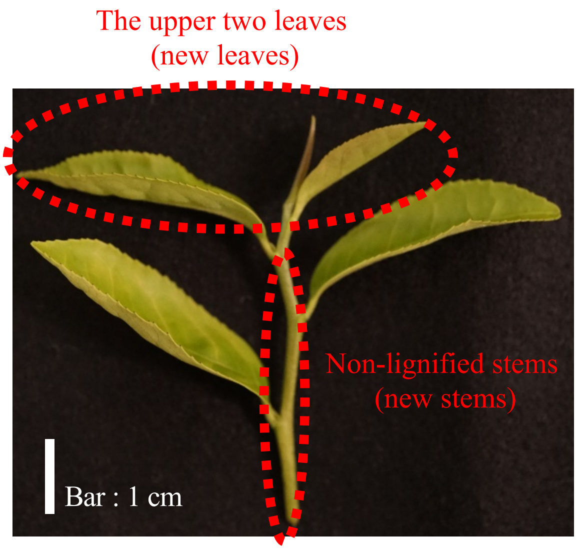

Supplementary Figure S1 New leaves and stems in first crop tea that was plucked for analysis in this study.

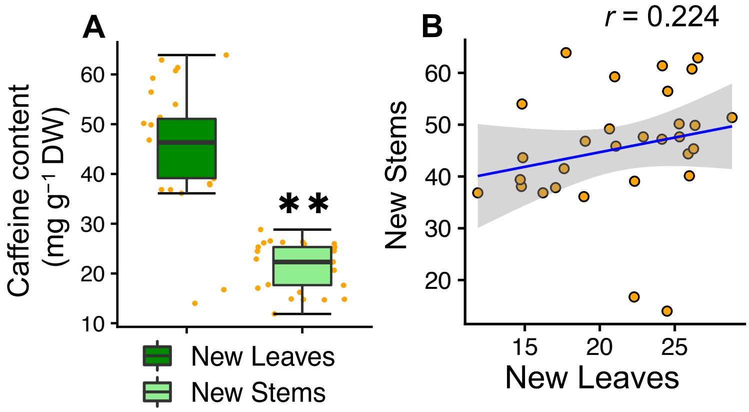

Supplementary Figure S2 Boxplot (A) and correlations (B) of caffeine content in new leaves and stems of the tea accessions. The double asterisks indicate $P < 0.01$ (Welch's t-test).

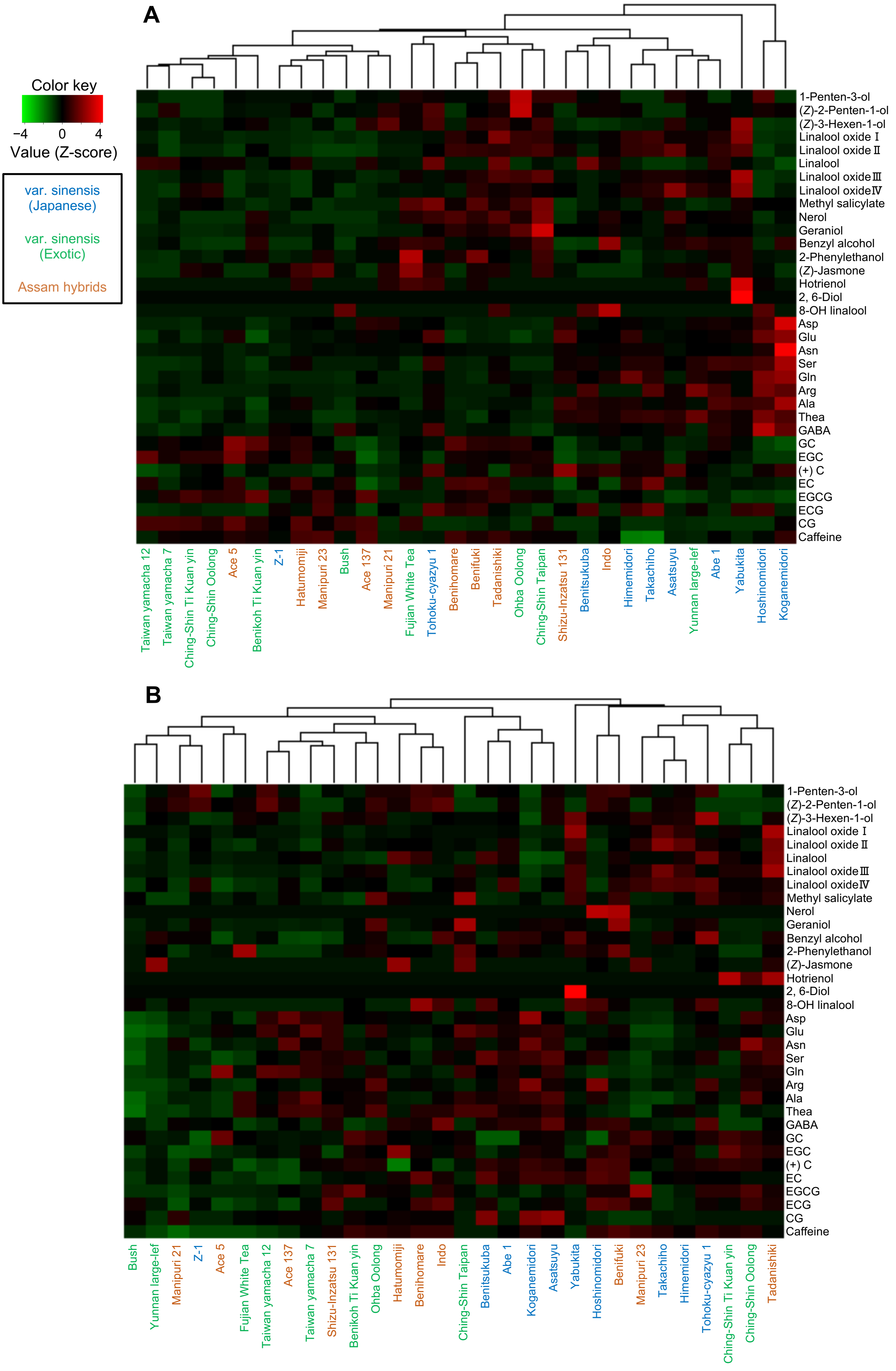

Supplementary Figure S3 Hierarchical cluster analysis of quantified quality-related metabolites in new leaves (A) and stems (B) of tea accessions.

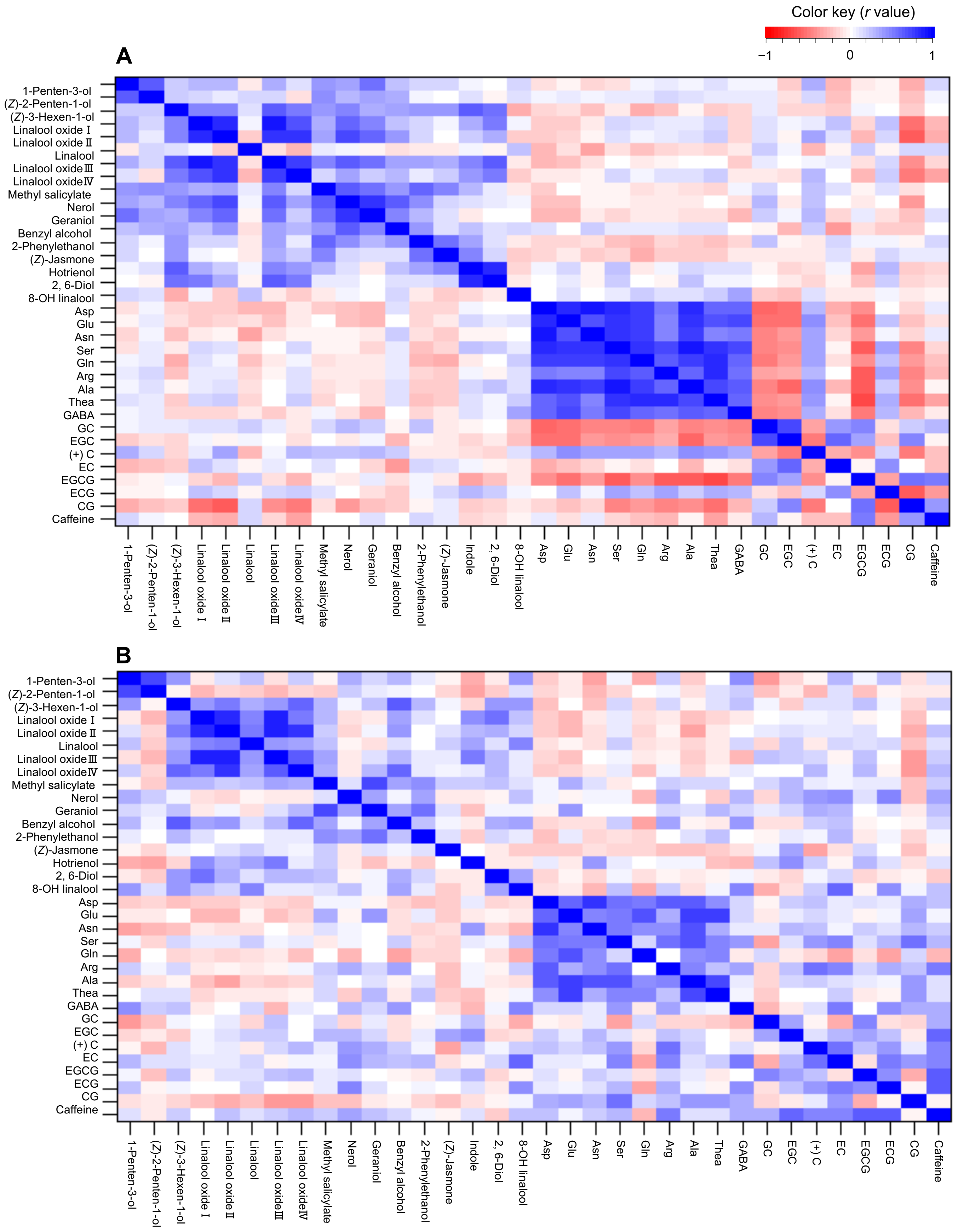

Supplementary Figure S4 Correlation plots of tea quality-related metabolites in new leaves (A) and stems (B). Different plot colors indicate major tea metabolites in each accession.

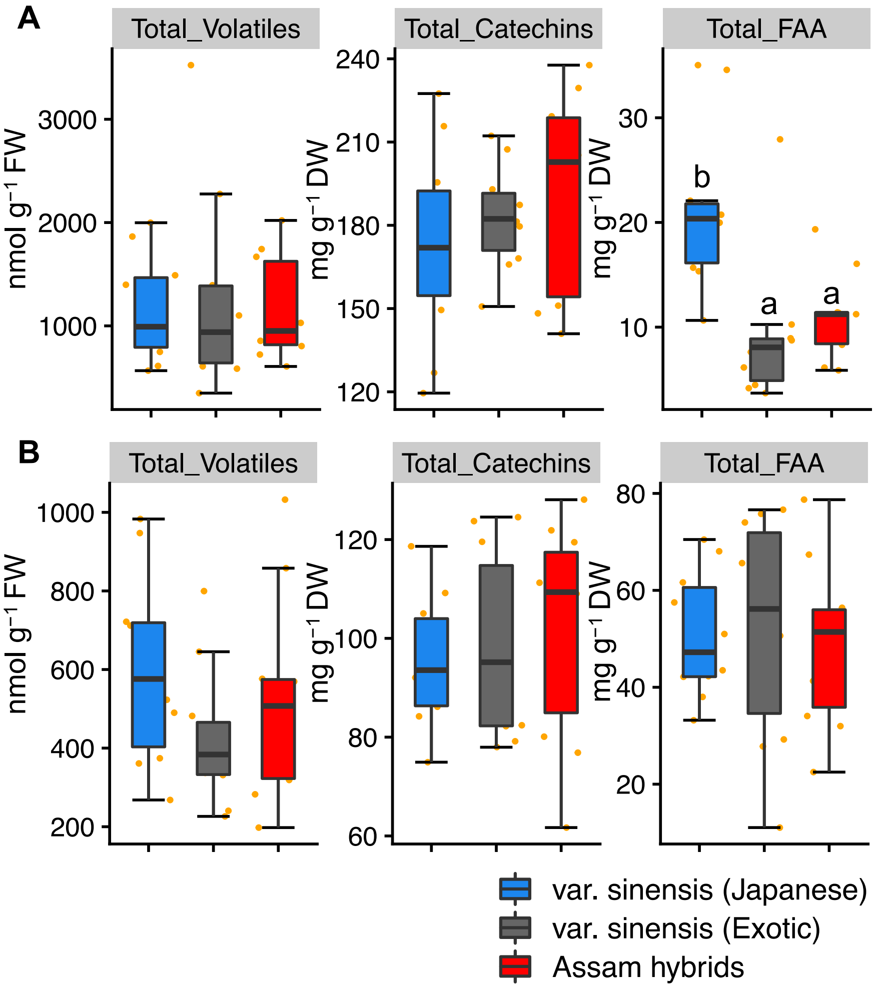

Supplementary Figure S5 Boxplots of total volatiles, catechins and amino acids contents in new leaves (A) and stems (B) of the tea accessions among genetic populations. Different letters indicate significant differences (Tukey's test, $P < 0.05$)

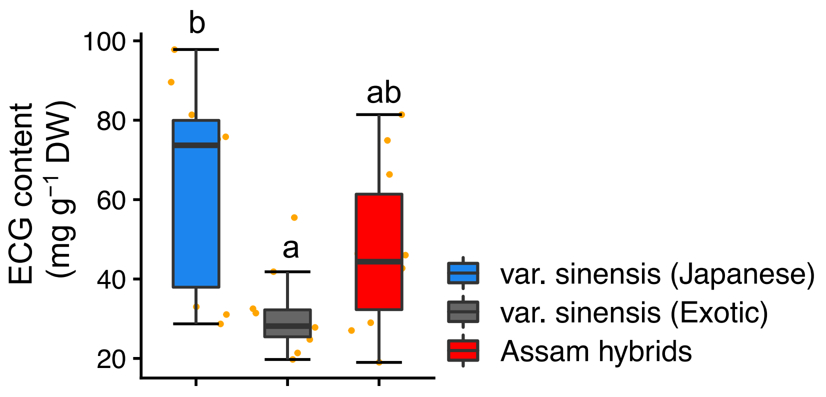

Supplementary Figure S6 Boxplots of ECG content in new leaves among genetic populations. Different letters indicate significant differences (Tukey's t-test, $P < 0.05$)

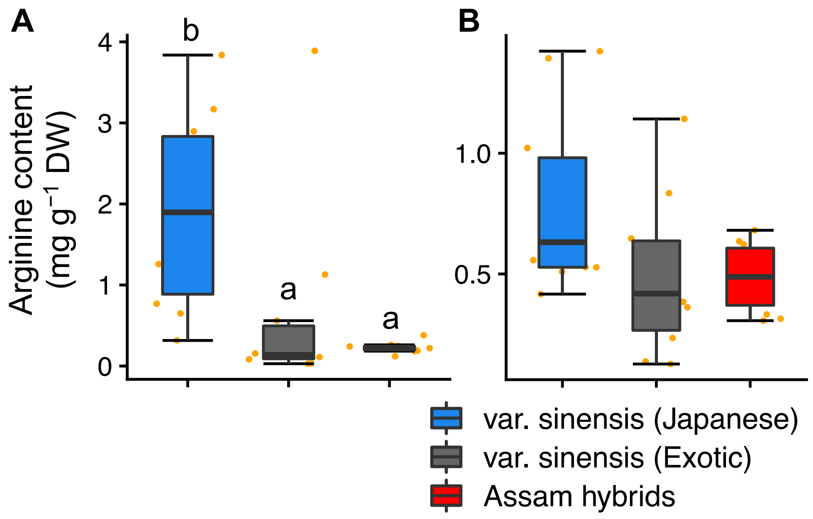

Supplementary Figure 7 Boxplots of arginine content in new leaves (A) and stems (B) among genetic populations. Different letters indicate significant differences (Tukey's t-test, $P < 0.05$)

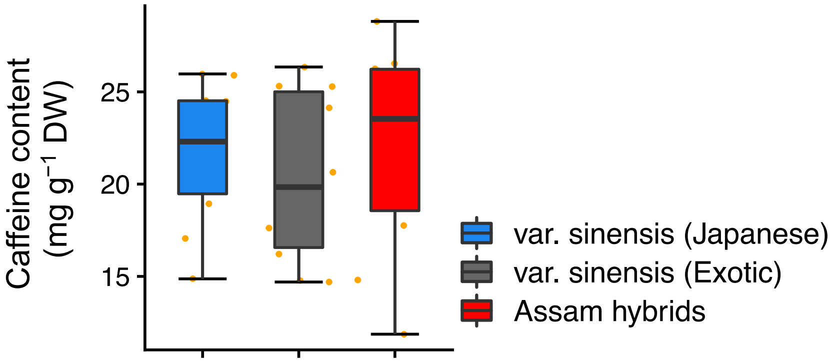

Supplementary Figure S8 Boxplots of caffeine content in new stems among genetic populations. Different letters indicate significant differences (Tukey's t-test, $P < 0.05$)
